# Brief research report: Chest radiographic thoracic area in term ventilated infants without respiratory disease

**DOI:** 10.3389/fped.2022.1042341

**Published:** 2023-01-09

**Authors:** Theodore Dassios, John Adu, Anne Greenough

**Affiliations:** ^1^ Neonatal Intensive Care Centre, King’s College Hospital NHS Foundation Trust, London, United Kingdom; ^2^ Women and Children’s Health, School of Life Course Sciences, Faculty of Life Sciences and Medicine, King’s College London, London, United Kingdom; ^3^ Department of Radiology, King’s College Hospital NHS Foundation Trust, London, United Kingdom; ^4^ National Institute for Health Research (NIHR) Biomedical Research Centre Based at Guy’s and St Thomas’ NHS Foundation Trust and King’s College, London, United Kingdom

**Keywords:** normal chest radiographic thoracic area newborn, infant, neonate, radiography, thoracic radiography

## Abstract

**Objective:**

To report values of the chest radiographic thoracic area (CRTA) in ventilated, term-born infants without respiratory disease and to evaluate whether CRTA is related to demographic data at birth.

**Methods:**

Retrospective, observational cohort study in a tertiary neonatal unit at King's College Hospital NHS Foundation Trust, London, UK.

Newborn infants born after 36 completed weeks of gestation, ventilated for poor perinatal adaptation or hypoxic ischaemic encephalopathy without respiratory disease and admitted in a recent eight-year period (2014–2022).

The CRTA was assessed by free-hand tracing of the perimeter of the thoracic area as outlined by the diaphragm and the rib cage excluding the mediastinal structures and was calculated using the Sectra PACS software.

**Results:**

One hundred and twenty-one infants (75 male) were included with a median (IQR) gestation of 40 (38–41) weeks and birth weight of 3.41 (3.04–3.75) kg. The median (IQR) CRTA was 2,589 (2,167–2,943) mm^2^ and was significantly related to birth weight (*r* = 0.316, *p* = 0.003), gestation at birth (*r* = 0.193, *p* = 0.032) and birth weight *z*-score (*r* = 0.187, *p* = 0.038).

**Conclusions:**

We report values of the chest radiographic thoracic area in ventilated term-born infants which could be used as reference for determining respiratory disease severity.

## Introduction

Quantitative assessment of the pulmonary parenchyma in neonatal intensive care has been undertaken by imaging and functional methods such as fetal magnetic resonance (1) or the measurement of the functional residual capacity (FRC) (2). An alternative and more accessible, surrogate method to estimate lung volumes is the chest radiographic thoracic area (CRTA), which is the measurement of the areas corresponding to the lungs on a two-dimensional chest radiograph. The CRTA has been shown to correlate well with the gold standard measurement of FRC by helium dilution (3). The measurement of the CRTA is important in diseases associated with decreased lung volumes. Low CRTA has been associated with failure to wean off invasive ventilation in preterm infants (4). The CRTA method has also been used in congenital diaphragmatic hernia (CDH) and predicted survival to discharge with an area under the receiver operator characteristic curve of 0.808–0.826 (5–7).

Although CRTA has been well-documented in pathological conditions, values in term infants without respiratory disease have not been reported. Such reference data would be important to establish the magnitude of the disease in respiratory disorders that are characterised by low lung volumes such as pulmonary hypoplasia due to prolonged preterm rupture of membranes (8) and diseases with hyperinflation such as bronchiolitis (9), severe asthma (10) or severe bronchopulmonary dysplasia (11). Our aim was to report values of CRTA in ventilated term-born infants without respiratory pathology and explore whether demographics at birth influence those values.

## Methods

### Subjects

Newborn infants treated for poor perinatal adaptation or hypoxic ischemic encephalopathy without concomitant respiratory pathology over 8 years (1/6/14–1/6/22) at King's College Hospital NHS Foundation Trust, London, UK were included in the study. The included infants were intubated and invasively ventilated for absence of respiratory drive at birth and had no supplemental oxygen requirement by 6 h of age. The starting point was selected as this was when the unit adopted volume-targeted ventilation as primary mode of mechanical ventilation. The infants were ventilated with a targeted tidal volume of 5 ml/kg and a positive end-expiratory pressure of 4 cm–5 cm H_2_O. The study was registered as a service evaluation with the Clinical Governance Department of KCH and, as it was not a research study, informed parental consent was not required. The following information was collected from the medical notes: sex, mode of delivery, gestation (completed weeks), birth weight (kg), birth weight *z*-score (12).

### Chest radiographs

**Figure 1:**
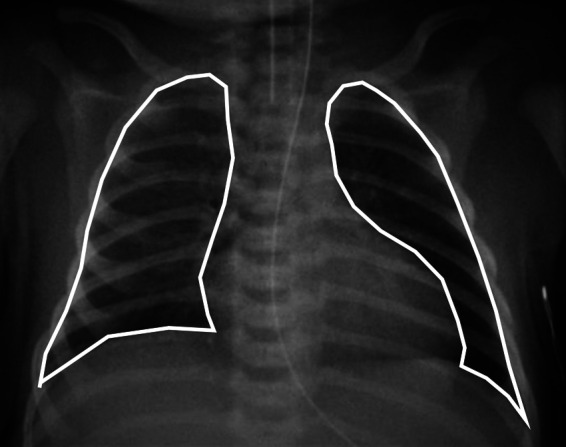
A chest radiograph of a term-born infant with poor perinatal adaptation on the first day of life. Method of free hand tracing of the perimeter of the CRTA excluding the mediastinal shadow.

The chest radiographs in the first 24 h after birth were reviewed for each infant, and the one with the highest CRTA was included in the analysis. The chest radiographs were anterio–posterior, obtained in the supine position at end-inspiration and at a standard distance of one metre above the infant. Rotated radiographs and radiographs with evidence of a pneumothorax or concomitant respiratory disease were excluded from the analysis. Rotation was assessed by measuring the distance between the medial edges of the clavicles to the vertebral spinous processes. The radiographs were imported as digital image files by Sectra PACS software (Sectra AB, Linköping, Sweden). The software automatically adjusted for magnification errors. Free-hand tracing of the perimeter of the thoracic area as outlined by the diaphragm and the rib cage was undertaken and the CRTA was calculated by the software (Figure 1). The repeatability of the method has been previously described with an inter- and intra-observer coefficient of repeatability of 1.06 cm^2^ and 1.0 cm^2^ respectively (3).

### Statistics

Data were tested for normality using the Kolmogorov–Smirnov test, found to be non-normally distributed and were presented as median (interquartile range). The relationships of CRTA with gestational age, birth weight and birth weight *z*-score were examined with the Spearman's *ρ* correlation coefficient. Differences in CRTA between male and female infants were assessed for statistical significance using the Mann-Whitney rank sum test. The relationship of the CRTA with birth weight was also examined with linear regression analysis to derive an equation to predict CRTA based on the birth weight. Statistical analysis was performed using SPSS software (SPSS Inc., Chicago IL).

## Results

**Figure 2:**
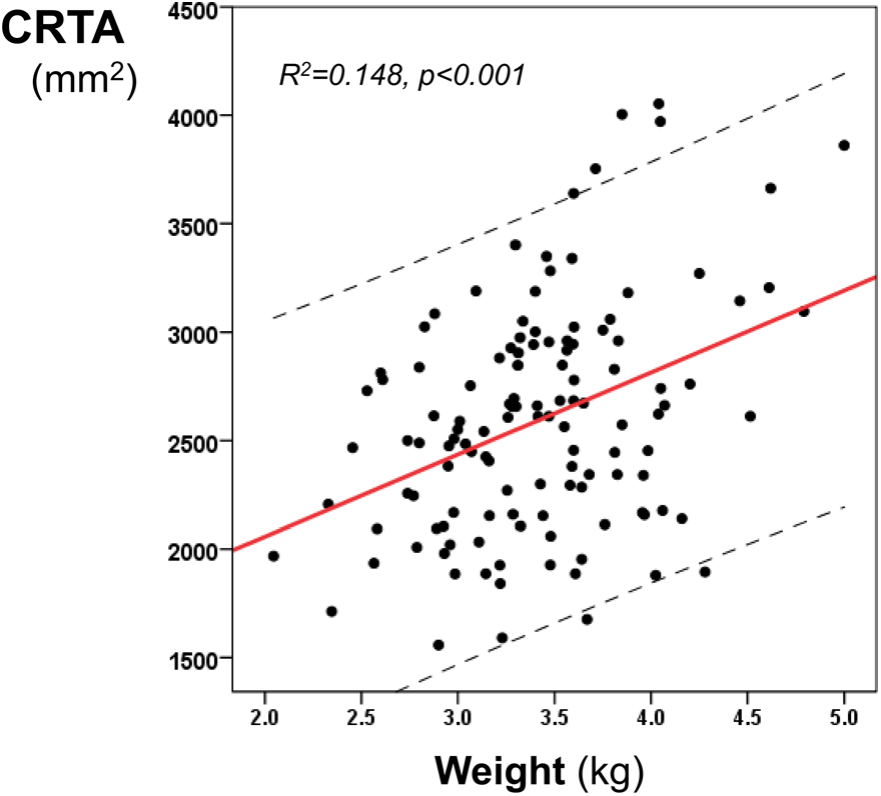
Linear regression analysis of the CRTA and the birth weight. The regression line and 95% confidence intervals (dashed lines) are depicted.

During the study period, 158 infants born after 36 completed weeks of gestation with poor perinatal adaptation or hypoxic ischemic encephalopathy were ventilated on the neonatal unit at KCH. Thirty-seven infants were excluded because of rotated radiographs, pneumothorax or concomitant respiratory disease. One hundred and twenty-one infants (75 male) were included with a median (IQR) gestational age of 40 (38–41) weeks, birth weight of 3.41 (3.04–3.75) kg and birth weight *z*-score of 0.03 (−0.59–0.75). The median (IQR) CRTA of the right lung was 1,486 (1,298–1,793) mm^2^ and of the left lung was 991 (852–1,202) mm^2^. The total CRTA was 2,589 (2,167–2,943) mm^2^ and was significantly related to birth weight (*r* = 0.316, *p* = 0.003 (figure 2)), gestational age (*r* = 0.193, *p* = 0.032) and birth weight *z*-score (*r* = 0.187, *p* = 0.038). The CRTA was not significantly different in male compared to female infants (*p* = 0.441). Following linear regression analysis, birth weight could predict the CRTA (*R*^2^ = 0.148, *p* < 0.001, 95% confidence intervals: 215–542). The regression equation was:

$$\mathrm{CRTA}\;\left(mm^{2}\right)=378\times \mathrm{BW}\;\left(\mathrm{kg}\right)+1300$$

## Discussion

We have reported CRTA values in ventilated term newborns without respiratory pathology. The CRTA was related to birth weight and gestation at birth. We report a median CRTA of 2,600 mm^2^ which is considerably higher than the 1,680 mm^2^ that we have previously reported in infants with CDH (7), reflecting that the latter infants had pulmonary hypoplasia. Amodeo and co-workers also studied 77 infants with CDH within 24 h after birth and reported that infants who died had lower CRTA values compared to the ones that survived, infants with hernia recurrence had lower CRTA and lower CRTA was associated with increased systolic pulmonary artery pressure (5). Weiss and co-workers measured the CRTA in 255 infants with CDH and demonstrated that the CRTA correlated significantly with survival and need for extracorporeal membrane oxygenation and that the CRTA displayed a higher ability to predict those outcomes compared with the widely used observed to expected lung/head ratio (6).

Other than in CDH infants, we have also reported that the median CRTA in 22 premature infants with bronchopulmonary dysplasia born at a median gestation of 26 weeks was 2,956 mm^2^, which is higher than our present study, but that result included the mediastinum, as in premature infants the mediastinal area sometimes cannot be clearly delineated from the lung fields (13). In our current study we reported that the ratio of the right to left lung CRTA is approximately 1.5. This might partially explain why unilateral right lung pathology is often clinically more severe than unilateral left, such as in right-sided CDH which has been associated with greater long-term morbidity (14), although clearly other factors might also play a role such as that the liver, which is herniating in right-sided CDH, is a non-compressible organ. In children and young adults, the volume of the right lung has been reported as 1.12 times larger than the volume of the left lung (15) and the weight as 1.14 times larger (16) but the projection of a three-dimensional structure to a two-dimensional radiograph might explain this discrepancy.

The correlation of the CRTA with the birth weight was significant, but arguably modest. This might be explained by the phase of breathing during which the radiograph was obtained. Although an effort is made to time the radiograph at end-inspiration, the high breathing rate of newborns might make this synchronisation challenging. There was, however, a clear separation of CRTA values in our study (interquartile range: 2,167–2,943 mm^2^), compared with infants with congenital lung pathology (interquartile range in CDH: 1,064–1,986 mm^2^) (7) which highlights that the possible impact of synchronisation on the CRTA would not affect the ability of CRTA to separate normal from pathological lungs. It is also possible that since the radiographs were performed on the first day of life in accordance with previous studies (5–7), some delayed clearance of lung fluid might be present in some infants (17). We should note that methodologically CRTA would be more appropriate to assess lung volumes in diseases that are characterised by lung hypoplasia, but would be of limited value for diseases with infiltrations such as pneumonia or neonatal acute respiratory distress syndrome. On the other hand CRTA would be expected to be increased in diseases that are characterised by hyperinflation or gas trapping such as emphysema or meconium aspiration syndrome respectively. In our population, the PEEP was kept relatively constant as per our internal protocol for term infants without respiratory disease. A higher PEEP, however, such as 6 or 7 cm H_2_O could affect the CRTA by producing artificially higher values.

Our study has strengths and some limitations. We included a large number of infants that were ventilated on volume-targeted ventilation. Infants ventilated for hypoxic ischemic encephalopathy are often receiving whole body hypothermia which might impact on respiratory function (18). Ventilation efficiency and tidal volumes have been reported to non-significantly increase during hypothermia (18), but in the era of volume targeted ventilation the effect of hypothermia on tidal volumes would be minimised.

In conclusion, we report values of the chest radiographic thoracic area in ventilated term newborn infants which could be used as a reference for determining respiratory disease severity in diseases that are characterised by lung hypoplasia.

## Data Availability

The raw data supporting the conclusions of this article will be made available by the authors, without undue reservation.
